# Why we believe chatbots: trust calibration as a design problem

**DOI:** 10.3389/fpsyg.2026.1935527

**Published:** 2026-09-08

**Authors:** Kokil Jaidka, Mengxuan Cai

**Affiliations:** 1 Centre for Trusted Internet and Community, National University of Singapore, Singapore, Singapore; 2 Department of Communications and New Media, National University of Singapore, Singapore, Singapore

**Keywords:** AI literacy, chatbots, engagement mechanisms, interpretability, large language models, trust calibration

## Abstract

Chatbots powered by large language models (LLMs), such as ChatGPT and Claude, answer questions quickly and fluently, yet they reveal little about how their answers are produced or what evidence supports them. Users tend to trust these systems based on surface qualities such as fluency, confidence, and speed. This trust is often miscalibrated, meaning that it does not match what the systems can actually do. Some users overtrust answers they cannot verify. Others distrust the systems and disengage. Large surveys show that many people rely on AI daily while saying they do not trust it. This article treats miscalibration as a design problem and develops a framework for recalibrating user trust. We first define trust in chatbots by connecting psychological research on trust, communication research on credibility, and human factors research on trust in automation. We then introduce a user typology built on two dimensions, the ability to verify chatbot outputs and the motivation to do so, which together predict how a given user will miscalibrate. On this basis, we synthesize two families of design intervention. Interpretability affordances, such as source citations and uncertainty cues, make evaluation possible. Engagement mechanisms, such as cooling-off periods and dialogue scaffolds, make evaluation happen by building it into the interaction. Following the Swiss cheese model of layered defense, we argue that these interventions cover one another’s gaps, and we state eight testable propositions that link each intervention to the users it serves. We close by discussing AI literacy as the slowest but most durable layer of defense.

## 1 Introduction

Chatbots powered by large language models (LLMs), such as ChatGPT and Claude, have become a primary gateway to information. Once confined to experimental applications, they now sit inside search engines, writing assistants, educational platforms, and customer service systems. Recent estimates indicate that nearly 80% of users rely on LLM-generated zero-click responses for at least 40% of their queries, and that more than half of search sessions end without a visit to any external website (Bain & Company, 2025). By mid-2025, ChatGPT alone was reaching roughly 700 million weekly users, about one in 10 adults worldwide (Chatterji et al., 2025). The appeal is easy to understand. These systems answer quickly, fluently, and across almost any domain, from education and healthcare to software development and legal guidance (Thirunavukarasu et al., 2023).

This shift changes the structure of information seeking, not simply its speed. A traditional search engine returns a ranked list of sources, and the user compares, evaluates, and triangulates among them. A chatbot collapses that work into a single synthesized answer, and often, the interface no longer shows users that alternatives exist. User behavior tracks this design: many users accept the first answer, ask fewer follow-up questions, and skip verification, as the zero-click figures above suggest. While users appear to accept the answer the chatbot synthesizes, whether acceptance amounts to perceiving chatbots as trustworthy authorities in a psychological sense remains an open empirical question.

Search behavior begins with the user’s own formulation, whether a keyword query or a conversational prompt. The consequences of a poor formulation differ sharply between the two. In a search engine, a badly framed query visibly returns poor results, and the user rephrases. In a chatbot, the wording, tone, and specificity of the prompt shape the content and the confidence of a single answer; however, the interface offers few signals that the framing was off. Research confirms both problems: model outputs change substantially with small, meaning-preserving changes in prompt format (Sclar et al., 2024), and non-expert users struggle to write and test prompts systematically (Zamfirescu-Pereira et al., 2023).

Whatever the quality of the framing, the fluency of the answer then does the persuading. Decades of research show that people believe smoothly presented information more readily, a tendency known as the fluency bias. Statements printed in easy-to-read fonts are judged more truthful than the same statements in harder fonts (Reber and Schwarz, 1999), and rhyming aphorisms are rated more accurate than non-rhyming ones with the same meaning (McGlone and Tofighbakhsh, 2000; see Alter and Oppenheimer, 2009, for a review). Chatbot responses are optimized for exactly these surface qualities, yet their fluency is decoupled from their accuracy. The jury is still out on whether users perceive the stylistic tropes typical of chatbot-generated text, such as its repeated formulaic expressions, phrases, and epithets (Kobak et al., 2025), as more or less trustworthy than text ostensibly written by humans; what is established is that readers cannot reliably tell the two apart, relying on intuitive but flawed heuristics (Jakesch et al., 2023).

Beyond stylistic tropes, chatbots have been reported to produce fabricated but convincingly formatted evidence, including medical references (Alkaissi and McFarlane, 2023) and legal citations (Dahl et al., 2024), and hallucination is documented across model families (Ji et al., 2023). These characteristics persist despite model upgrades, in part because of how these systems are trained and evaluated. Standard evaluations reward confident guessing over admissions of uncertainty (Kalai et al., 2025), so chatbots may deliver wrong answers in the same assured register as correct ones, leaving users no linguistic cue to tell them apart. Likelihood-based training and alignment tuning favor high-probability continuations, so models converge on safe, formulaic phrasings instead of high-entropy text. And the difficulty of attributing outputs to specific training data helps explain why most systems reveal little about how an answer was produced or what evidence supports it (Liu et al., 2023). As a result of these and other design choices, users are given strong cues to believe and weak means to check.

This diagnosis is not overturned by the transparency features that mainstream chatbots have begun to add. Search grounding, inline citations, and expandable source panels are spreading, and the trajectory points toward more disclosure, not less. Yet disclosure has not, by itself, recalibrated trust. Behavioral data confirm that sources are shown but seldom consulted. In a passive tracking study of 900 United States adults, users who encountered an AI-generated summary clicked a cited source within it in only 1% of visits, and clicked any result link about half as often as users who saw no summary (Chapekis, 2025). The pattern is what a design analysis predicts: transparency features are passive affordances that wait for the user to act, and the users most at risk of miscalibration are the least likely to act on them. The problem this article addresses is therefore the inertness, not the absence, of transparency; disclosure alone cannot close the gap between the cues users are given to believe and the means they have to check.

Some scholars call this phenomenon the AI trust paradox, in which users place high trust in systems that offer little transparency or accountability (Jiang et al., 2026). We advise caution with this label: people routinely trust systems they cannot verify, such as medicines or air travel, and that is normal cognition, not a contradiction. The chatbot case differs in two ways. First, trust in medicine or aviation rests on institutional safeguards such as regulation, licensing, and audit. Trust in chatbot outputs has no comparable warrant, because the cues users rely on come from the same system that generates the content and carry no independent information about accuracy. Second, users often know this. In a study of over 81,000 people across 159 countries, unreliability was the single most cited concern about AI, yet the same respondents valued AI for decision support (Huang et al., 2026). A global survey of over 48,000 people across 47 countries shows the same dissociation: two thirds reported using AI regularly, while less than half were willing to trust it (Gillespie et al., 2025). Heavy use therefore reflects convenience more than confidence, which means the ‘high trust’ the paradox posits may be an artifact of equating reliance with trust.

We therefore describe the problem as trust miscalibration rather than as a paradox. Trust is calibrated when its level matches what the system can actually do (Lee and See, 2004). In chatbot use, trust is decoupled from that standard in both directions. Some users overtrust confident but unverifiable answers, the pattern the automation literature calls misuse. Others disengage entirely, the pattern known as disuse (Parasuraman and Riley, 1997). Both failures are costly in domains such as healthcare, law, and education, where the appearance of reliability can mask substantive error.

This article argues that miscalibration is a design problem before it is a user problem, and that interpretability should be a structuring principle of chatbot design, not a feature added afterwards. Drawing on human–computer interaction, psychology, and communication research, we make four contributions. First, we ground the argument in a conceptual framework that connects trust, credibility, and calibration, and we state a working definition of trust in chatbots. Second, we introduce a user typology built on two dimensions, the ability to verify outputs and the motivation to do so, which specifies who each design intervention is for. Third, we synthesize two families of intervention. Interpretability affordances make evaluation possible, and engagement-based incentive mechanisms make it happen. The mechanisms adapt intervention logic from misinformation and nudging research, and we argue that the conversational setting changes what this logic can do. Fourth, we state eight testable propositions that make the framework accountable to evidence.

The article proceeds as follows. We first define trust and calibration, then show how chatbot design pushes trust away from calibration in both directions, and then introduce the user typology. Two design sections follow, one on interpretability affordances and one on engagement mechanisms. Throughout, we use Reason’s (1990) Swiss cheese model as the integrating frame, in which no single layer of defense suffices but the layers cover one another’s gaps. We close with the formal propositions and with user literacy as the slowest but most durable layer of defense.

## 2 Conceptual foundations of trust in human–AI interaction

We define trust in a chatbot as a user’s willingness to accept and act on its outputs without independent verification. This willingness is an attitude, and actual reliance is its behavioral expression; trust is calibrated when the willingness matches the actual accuracy and verifiability of the outputs. Later sections mention variants such as instrumental trust, and in every case we mean this same willingness shaped by a particular motive or setting. The definition condenses four bodies of research, which this section reviews in turn.

Psychological research provides the first anchor for our framework. Mayer et al. (1995) define trust as the willingness to be vulnerable to the actions of another party. The trustor expects the other party to act in their interest, yet cannot monitor or control what that party does. Rousseau et al. (1998), comparing definitions across disciplines, similarly conceptualize trust as a psychological state in which a person accepts vulnerability because they hold positive expectations about another’s behavior. Both definitions imply that trust becomes relevant only when the trustor faces uncertainty and something is at stake. A chatbot user meets both conditions, as the user cannot inspect how an answer was produced (Liu et al., 2023), and a wrong answer may bear serious personal or societal consequences. These definitions also separate trust from two neighboring concepts that are easy to confuse. Trustworthiness is a property of the trusted party, which Mayer et al. (1995) describe through ability, benevolence, and integrity. Trust itself is an attitude. Reliance is the behavior that may follow from that attitude.

Lee and See (2004) argue that the difference between trust and reliance is essential in human–machine settings. People sometimes rely on a system they claim not to trust, and sometimes refuse to rely on a system they rate highly. Much of the evidence reviewed in this article measures reliance, for example accepting an answer without checking any source. We treat such behavior as the observable footprint of trust, not as trust itself. Reliance without verification carries real costs, and these are increasingly coming to light: a patient was hospitalized for weeks with bromide poisoning after acting on a chatbot’s dietary suggestion (Eichenberger et al., 2025), and fabricated legal citations generated by chatbots have been filed in real court proceedings (Dahl et al., 2024). It is perhaps no wonder that trust and reliance have drifted apart: two thirds of respondents use AI regularly while less than half say they are willing to trust it (Gillespie et al., 2025).

The credibility literature in communication research provides a second anchor for the framework. In the classic source credibility tradition, receivers judge a communicator on two dimensions: expertise captures whether the source knows the truth, and trustworthiness captures whether the source will tell the truth (Hovland and Weiss, 1951; O’Keefe, 2002). Both dimensions map onto our argument, and in both cases the judgment process matters as much as the judgment itself. People seldom assess expertise through careful analysis; they rely instead on fast heuristic cues such as professional design or a confident tone (Metzger and Flanagin, 2013), which is also how users come to judge a chatbot as knowledgeable. The trustworthiness dimension is harder still, because users can only judge whether a source tells the truth when they can check its claims. A credible source in the classic sense invites verification, which is something that current chatbots rarely offer.

Lee and See (2004) argue that the goal of design is not to maximize trust but to calibrate it. Trust is calibrated when its level matches what the system can actually do. Calibration may fail in two directions: on the one hand, users may trust too much and apply automation in situations it cannot handle, which Parasuraman and Riley (1997) call misuse. On the other hand, users may also trust too little and abandon automation that would have served them well, a pattern the same authors call disuse. Yet, it is also important to consider which human factors predict higher trust in automation. Hoff and Bashir (2015) reviewed more than 100 studies and concluded that trust in automation forms on three layers: dispositional trust reflects stable individual traits, situational trust reflects the task and context, and learned trust reflects experience with the specific system. These layers are discussed later in this article to explain why the same design intervention does not work equally well for every user.

Finally, computing research offers the third foundational anchor to support our framework. One strand of this literature treats trust as a quantity that one system computes about another, from Marsh’s (1994) formal foundation to later surveys of networked and multi-agent systems (Cho et al., 2015; Grandison and Sloman, 2000). We set that strand aside, because our trustor is a person, not a machine, and draw instead on research concerning human trust in intelligent systems. Andras et al. (2018) argue that as machines become more autonomous, trust has to be built into the socio-technical system around them, and it cannot be assumed. Jacovi et al. (2021) clarify that trust is warranted only when it is caused by the actual trustworthiness of the model, and unwarranted when it rests on other cues—the computing counterpart of calibration. Vereschak et al. (2021) add a methodological warning that we return to when stating our propositions: studies of AI-assisted decision making often measure trust in ways that do not match their own definitions. With the definition in hand and its components anchored, the next section argues that the interface and interaction style of current chatbots push trust away from calibration in both directions at once.

## 3 Miscalibrated trust in AI chatbots: fluent but unverifiable

Despite their opaque workings, chatbots increasingly command user trust. This trust rarely stems from a deep understanding of how these systems work. It stems from surface cues such as fluency, confidence, and responsiveness. In the sense defined above, such trust is miscalibrated. It tracks the polish of the response rather than the properties that make a system trustworthy, such as accuracy and verifiable sourcing (Lee and See, 2004). As chatbots generate increasingly natural and context-sensitive responses, users become more likely to treat their outputs as authoritative, even in the absence of sourcing or methodological transparency (Steyvers et al., 2025). We first examine why many users trust too much, then why others withhold trust altogether, and finally what the two failures share.

The first failure of calibration is overtrust, and its psychological basis is well established. The Computers as Social Actors (CASA) framework posits that people apply human social heuristics to computers when they exhibit human-like behavior (Lee and Nass, 2010; Nass et al., 1994). Conversational fluency activates such heuristics. Users interpret politeness, coherence, and responsiveness as signs of competence. These cues reduce critical scrutiny (Sundar, 2004), leading users to conflate how something is said with whether it is true (Ahn et al., 2021). The persuasive delivery style of chatbots thus raises perceived reliability while the underlying process stays hidden.

Overtrust is also affectively reinforced: many users describe chatbots as “empathetic,” “nonjudgmental,” or “understanding” in emotionally charged contexts (Lucas et al., 2014). In domains like mental health, peer support, or personal decision-making, users turn to chatbots not for factual correctness but for affective reassurance (Abd-Alrazaq et al., 2019). Fitzpatrick et al. (2017) found that users of a mental health chatbot reported emotional attachment and wellbeing benefits despite having no means of verifying its responses. Skjuve et al. (2021) documented users forming genuine companionship bonds with chatbots irrespective of output accuracy. This social and emotional resonance deepens trust and creates a sense of psychological safety even when outputs are unverified (Sundar, 2020).

Empirical studies confirm these dynamics. Users frequently overestimate the correctness of LLM outputs when responses are expressed confidently or elaborately (Steyvers et al., 2025). This tendency, described as algorithmic complacency (Basu and Dave, 2024), reflects a substitution. Rather than assess factual accuracy, users lean on affective and stylistic cues. The result is overtrust in outputs that may be fabricated or incorrect, especially when users lack domain expertise. Machine authorship can even strengthen this effect. Markowitz (2024) compared summaries of scientific papers and found that readers rated the scientists as more credible and more trustworthy when the summaries were written by GPT instead of by humans, largely because the model wrote in simpler language. Experimental evidence ties this substitution to the conversational format itself. In a preregistered experiment in the United States and India, participants who explored political information through chatbot interfaces reported greater confidence and larger knowledge gains than participants given a static list of results, yet those using a GPT-4o-based search pipeline became less accurate at distinguishing misinformation, and they rated its demonstrably less relevant and more controversial retrieval as no less credible than a conventional aggregator (Jaidka et al., 2026).

There is also a system-side reason for this decoupling. Alignment and safety training shapes chatbot output toward language that is polite, balanced, and acceptable, but acceptability is not accuracy. Philosophers have pressed this point. Hicks et al. (2024) argue that model output is best understood as bullshit in Frankfurt’s sense, produced with indifference to truth, not with an intent to deceive. Licon (2025) traces part of this indifference to the human discourse the models learn from. Muttaqin et al. (2025) describe the result as a form of sophism, persuasive in style rather than sound in substance. For our argument the implication is that the layer which makes a chatbot sound trustworthy is not the layer that makes it correct, so users receive a stronger quality signal than the system can justify.

Importantly, overtrust is context-sensitive. In high-stakes domains such as medicine or law, users are more likely to triangulate chatbot outputs with external expertise or seek human verification (Nori et al., 2023). Expert review of GPT-4’s answers to medical licensing questions shows that fluent responses can contain errors that only domain knowledge reveals (Kung et al., 2023), and legal experts examining ChatGPT-generated responses detected coherence gaps that non-experts were likely to overlook (Choi et al., 2023). In low-stakes settings such as grammar correction or trivia, fluency is more readily equated with accuracy (Jakesch et al., 2023). This pattern aligns with the distinction between fast, intuitive thinking and slower, analytical thinking (Kahneman, 2011). Low-stakes queries invite the former, and high-stakes contexts trigger the latter. The Elaboration Likelihood Model makes a similar prediction. Users with low involvement process information through peripheral cues such as fluency and confidence instead of engaging critically with content (Petty and Cacioppo, 1986). Platform context also shapes expectations. Trust in a familiar platform can transfer to the chatbot embedded in it, so users on institutional or branded platforms often assume a baseline of curation or oversight (Stewart, 2003).

The second failure of calibration is distrust. Some users disengage from chatbots altogether and may reject even accurate outputs (Shen, 2022). This skepticism reflects a protective rationality. When verification is impossible, trust becomes untenable. Yet disengagement carries its own costs. It can cut users off from beneficial AI support and reinforce digital divides between those who adapt and those who opt out (Jacovi et al., 2021).

The two failures do not belong to two separate groups of users. They often coexist within the same person. In a scoping review of university students’ experiences with generative AI, Gow and Illingworth (2026) identified dynamic tensions between productivity and dependency, emotional empowerment and cognitive offloading, and epistemic agency and uncritical reliance. Students valued AI for reducing task friction and providing nonjudgmental support, yet they simultaneously recognized risks of diminished critical thinking and over-dependence. Large-scale survey data show the same pattern. Huang et al. (2026) found that respondents who valued AI for emotional support were approximately three times more likely to also report concerns about emotional dependency. These tensions mirror trust miscalibration at the level of the individual user.

Users do try to cope with this opacity, but their strategies have clear limits. Common tactics include prompting the chatbot for citations, cross-checking answers in a search engine, and reading surface features such as response length or emotional tone as signs of credibility (Basu and Dave, 2024; Jacovi et al., 2021; Zamfirescu-Pereira et al., 2023). Whether these tactics help depends on who applies them and when. Digital literacy, domain expertise, and time pressure all shape whether a check happens and whether it succeeds (Frenkenberg and Hochman, 2025). Under pressure or in emotionally sensitive moments, even careful users fall back on fluency and choose reassurance over rigor (Finucane et al., 2000). We return to these individual differences later in the article. The point here is simpler. User effort alone cannot close a gap that design has created.

Social and institutional pressures further reinforce this miscalibration. In many workplaces and classrooms, the imperative to adopt AI and to keep up leads to performative use and instrumental trust, even when confidence is low (Frenkenberg and Hochman, 2025). Instrumental trust here is still the willingness defined earlier, but users grant it for social reasons, not evidential ones. Over time, habituation to fluency without scrutiny can normalize shortcuts in how people evaluate information. This concern is amplified by evidence that educators are 2.5 to 3 times more likely to report witnessing cognitive atrophy among AI users than cognitive growth (Huang et al., 2026). Importantly, miscalibrated trust is not merely individual. Ashery et al. (2025) find that LLM users often converge on shared assumptions or biases, even when prompts are neutral. These emergent conventions render misinformation harder to contest, embedding flawed epistemic norms into collective practice. Similar concerns are now being raised about scholarship itself. Metcalf (2026) warns that widespread AI-assisted writing can erode a field’s knowledge base through small, individually harmless increments, and Muttaqin et al. (2026) link this to publication incentives that reward volume over contribution. These are the institutional counterparts of the individual habituation described above.

In sum, the growing trust in chatbots conceals a deeper problem. Fluency and convenience earn the trust, yet users are asked to believe systems they cannot interrogate or verify. This gap fuels both overtrust and avoidance. Without appropriate system designs, user trust will remain grounded in fluency and not in verifiability, which weakens critical engagement and undermines democratic knowledge practices (Chen, 2025; Pozzi, 2023). Recalibrating user trust requires disaggregating its two components, reliability and interpretability, and rethinking what trustworthy AI should entail in public knowledge systems.

## 4 Individual differences in trust calibration

Trust calibration does not fail in the same way for every user. The previous section located both failures in a single design choice; this section adds the user side of the equation, because the same opaque interface pushes different users in different directions. For instance, a cross-country study reported that conversations with chatbots narrowed the gender gap in political knowledge while widening it in misinformation discernment (Jaidka et al., 2026). We argue that two dimensions organize these differences: the ability to verify chatbot outputs and the motivation to do so. Crossing them yields four user profiles, each with a distinct calibration risk, and the design interventions developed in the rest of the article are matched to these profiles.

The two dimensions have a basis in the trust literature. Hoff and Bashir (2015) distinguish three layers of trust in automation: dispositional trust reflects stable traits, situational trust reflects the task and context, and learned trust reflects experience with the specific system. For chatbot use, we collapse these layers into two functional dimensions. Expertise and AI literacy accumulate through experience, so the learned layer mainly determines whether a user can verify an output. Stable traits such as the enjoyment of thinking shape whether a user *will* try, so the dispositional layer mostly feeds motivation. Ability and motivation, in combination, predict the dominant calibration risk, as they moderate whether a message is processed centrally or peripherally (Petty and Cacioppo, 1986). The situational layer acts on motivation from moment to moment; a profile is therefore a position, not an identity, and the same person may occupy different cells across tasks or even within a session.

The ability to verify rests on domain expertise and AI literacy. Domain experts can check an output against their own knowledge: medical evaluators found errors in fluent GPT-4 answers that domain knowledge was needed to detect (Kung et al., 2023), and legal experts detected coherence gaps that non-experts overlooked (Choi et al., 2023). Non-experts, by contrast, approach chatbots by trial and error and struggle to test them in any systematic way (Zamfirescu-Pereira et al., 2023). AI literacy works differently, by giving users a working model of what a language model is and why it can fabricate, which turns vague unease into targeted checking (Long and Magerko, 2020; Ng et al., 2021). Users who lack both resources are not passive, but their efforts tend to misfire.

Motivation to verify is a separate resource, and ability does not guarantee it. Some people enjoy effortful thinking more than others, a trait known as need for cognition (Cacioppo and Petty, 1982). Users high in this trait are more likely to process a response centrally, while users low in it default to peripheral cues such as fluency (Petty and Cacioppo, 1986). Epistemological beliefs matter as well: users who see knowledge as certain and handed down by authorities have little reason to question a confident answer (Hofer and Pintrich, 1997). Motivation is also the layer that context moves most easily. Time pressure, emotional load, and workplace expectations can suppress checking even among users who would otherwise engage in it (Finucane et al., 2000; Frenkenberg and Hochman, 2025).

**Figure 1:**
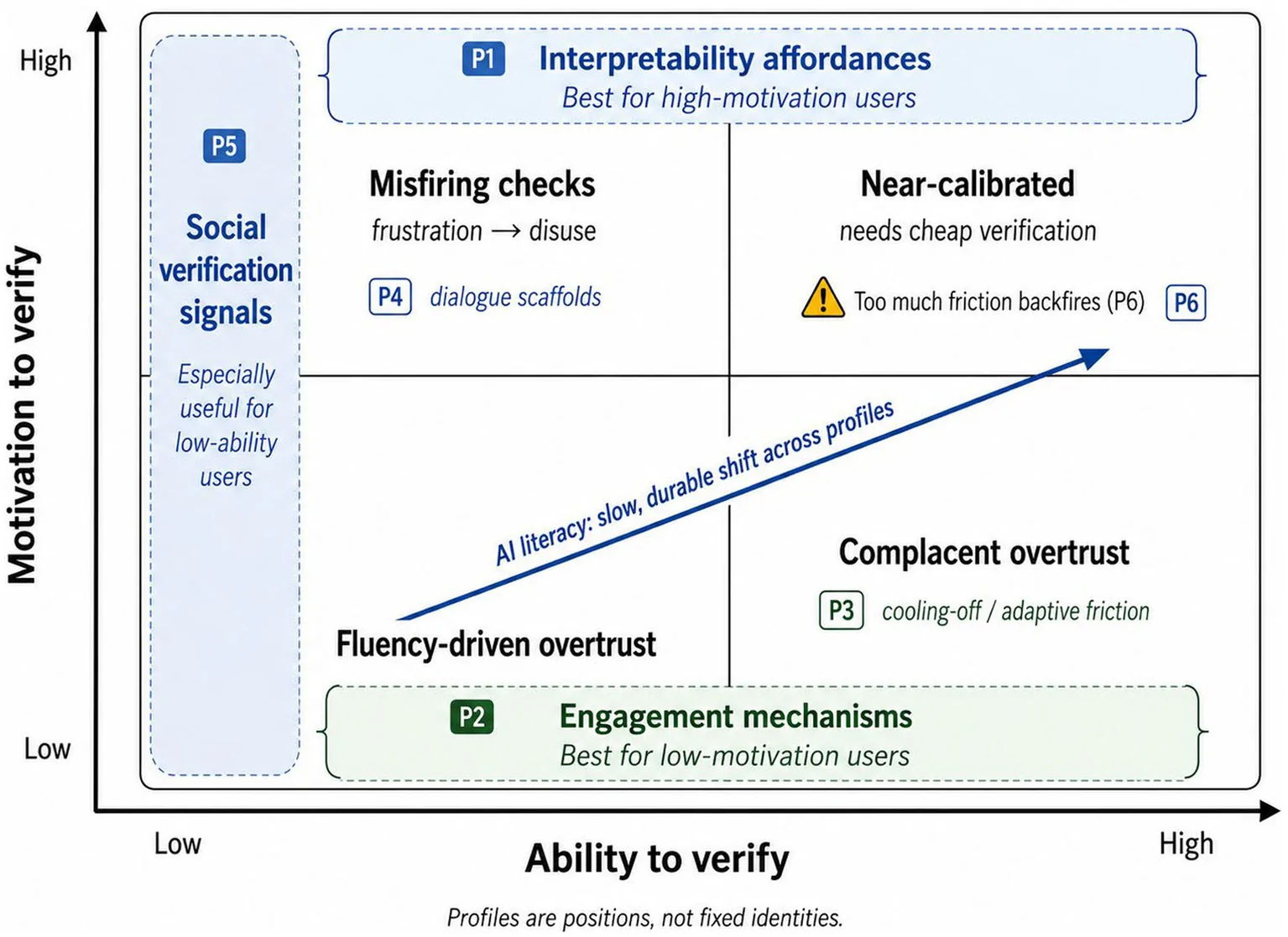
User-centered map of trust-calibration interventions. Ability and motivation to verify define four positions associated with different calibration risks. Interpretability affordances primarily support motivated users, engagement mechanisms address failures associated with low motivation, and social verification signals are especially relevant when verification ability is low. AI literacy operates differently by gradually increasing users’ capacity to verify and potentially moving them across profiles. P1–P6 refer to the formal propositions stated in Section 7.1.

These two dimensions are the axes of our typology (Figure 1). Crossing them yields four user profiles with distinct calibration risks. Users high in both ability and motivation come closest to calibrated trust, and they mainly need efficient verification tools, not persuasion. Users with high ability but low motivation know how to check and rarely do, so their risk is complacent overtrust, especially under time pressure. Users with low ability but high motivation want to check and cannot do it well; their effort produces either misplaced confidence in weak checks or frustration that ends in disuse. Users low in both dimensions are the most exposed to overtrust, because fluency is the only signal available to them. The typology is deliberately simple and the boundaries between profiles are soft, yet it gives design a target. The question is not simply whether an intervention works, but for whom. Figure 1 summarizes this user-centered logic by mapping each calibration risk to the intervention families most likely to address it. It also shows how AI literacy differs from the other interventions by moving users across profiles instead of acting only within a given profile. This typology reframes the design task that occupies the rest of the article.

## 5 Design directions: enhancing interpretability as affordance

Interpretability in chatbot design is a normative challenge, not only a technical one. We propose that interpretability in public-facing AI should serve three goals. First, it should protect the user’s ability to judge, so that users can tell established knowledge from speculation instead of treating all outputs as equally reliable (Chen, 2025). Second, it should make the system contestable. Users need to see enough of the inference path to question it and to give feedback, particularly in high-stakes settings (Ananny and Crawford, 2018). Third, it should preserve usability. The fluency that draws users in must survive the added transparency, or the design will simply be ignored (Shin, 2021). These goals connect interpretability to larger questions of information quality and democratic accountability.

To pursue these goals, interpretability must live in the interaction itself, not in backend mechanisms. In human–computer interaction and design theory, affordances are the possibilities for action that a system offers its user, what it invites, permits, or discourages (Gaver, 1991; Norman, 1999). Applied to chatbots, this lens treats the interface as the place where trust is calibrated or miscalibrated. Seen through this lens, current chatbot interfaces afford the wrong things. When a chatbot presents a polished, standalone answer with no signal of uncertainty, it affords finality and suggests that no further engagement is needed (Jacovi et al., 2021; Kim et al., 2024). When it draws no visual line between generated and retrieved content, it affords passive acceptance. When disclaimers and citations sit buried in footnotes, it affords neglect (Ananny and Crawford, 2018; Shen, 2022). This opacity can, on the one hand, leave engaged users with nothing to judge but the surface of the response, so their trust inflates. On the other hand, it can leave cautious users with no way to perform the verification that trust would require, so their trust collapses. Therefore, a single design misstep can produce both miscalibrations.

Yet, a single transparency feature cannot serve diverse user profiles. Expert users under time pressure need interventions that lower the cost of checking. Motivated novices need interventions that make their checks effective. Unmotivated users need interventions that create a reason to check at all. Therefore, we propose that interpretability can be built into the interaction through five strategies. Each serves the three goals in a different way, and each has a boundary that the user typology helps to locate.

The first strategy is source transparency, which shows users where the content of a response comes from. The simplest form is the inline citation, a reference attached to a specific claim inside the answer, so that each statement can be traced to its origin. Retrieval-augmented generation (RAG) goes deeper and changes how the answer is produced. The system first retrieves documents from a defined collection and then generates its response from those documents, so citations point to real texts instead of patterns the model absorbed during training (Huang and Chang, 2024; Ji et al., 2024). A third form is the confidence-calibrated hyperlink, a link annotated with signals of recency or reliability that help users decide which sources deserve attention. The timing of attribution shapes its quality. Pre-hoc methods choose sources before the answer is generated, which keeps the text aligned with evidence but restricts what the model can say. Post-hoc methods attach sources after the text is written, which is flexible but invites mismatches and fabricated references (Liu et al., 2023; Tilwani et al., 2024). Citation is therefore not a panacea. Fabricated or outdated sources may damage trust more than no sources at all, especially when they imitate academic formats. Alkaissi and McFarlane (2023) found that ChatGPT produced non-existent but convincingly formatted medical references. The typology marks the boundary of this strategy. Visible citations mainly serve users who are motivated to check. For users who cannot evaluate a reference, a fabricated citation is worse than none, because it affords misplaced confidence (Shen et al., 2025).

The second strategy is rationale generation, in which the system explains why it answered as it did. The explanation can take the form of visible intermediate reasoning steps, produced through chain-of-thought prompting, or a short account displayed next to the answer, such as a “Why this answer?” overlay (Ji et al., 2024). Early examples exist. Model cards describe a system’s capabilities before interaction begins (Mitchell et al., 2019). Slack’s AI summaries state which sources informed them, and Bing Chat offers expandable citation panels that let users trace claims to documents (Liao and Vaughan, 2024). The strategy carries a known risk. Generated rationales are themselves model outputs, and they may not reflect the computation that actually produced the answer (Jacovi and Goldberg, 2020; Papenmeier et al., 2019; Ribeiro et al., 2016). We take a clear position on this problem. Rationale generation is worth deploying, but under two conditions. The rationale must be paired with source attribution, so that its claims can be checked against something outside the model. And the interface must present the rationale as a useful account and not as a faithful report of internal processing, so that the explanation does not become one more fluent surface that inflates trust. Under these conditions, an imperfect rationale still gives users a concrete object to question, and questioning is precisely the behavior that calibrated trust requires.

The third strategy is uncertainty expression, which tells users how confident the system is in a given output. The signal can be verbal, such as the hedge “I am not sure,” numeric, such as a confidence score shown beside a claim, or contextual, such as a domain-specific disclaimer (Kim et al., 2024; Yona et al., 2024). The mechanism is simple. Models hold internal probability information that users never see, and uncertainty expression converts part of that hidden state into a visible cue that marks where checking is worthwhile. The evidence is encouraging. First-person hedges reduce overreliance more effectively than generic third-person disclaimers (Kim et al., 2024). Liu et al. (2024) showed that models can learn to hedge in low-confidence cases while staying assertive where confidence is warranted, and Kadavath et al. (2022) found that large models can be trained to evaluate the correctness of their own outputs. The signal itself must be calibrated, however. Excessive hedging erodes perceived competence, and overconfident language hides limitations (Mielke et al., 2022). The typology again marks the boundary. An uncertainty cue helps the users who read it, and motivated users do. Users who skim in a hurry may never register the hedge at all.

The fourth strategy is interactive scrutiny, which lets users interrogate an output instead of merely reading it. Basic tools include reviewing conversation history, highlighting the parts of the prompt that shaped the response, and posing meta-questions such as “How do you know that?” (Liao et al., 2020; Zhang et al., 2022). Richer tools show which earlier turns of the conversation influenced the current answer (Donkers et al., 2020; Liao and Vaughan, 2024), or let users request alternatives to a generated answer, such as dissenting viewpoints or contrasting citations (Chen, 2025). These tools turn interpretability from a static disclosure into an ongoing dialogue, which is what contestability requires (Jacovi et al., 2021). Their effect depends on legibility and visibility, not mere availability (Ananny and Crawford, 2018). They also reward users who already have both the ability and the motivation to interrogate the system, and they do little for anyone else.

The fifth strategy, progressive disclosure, addresses the typology most directly, because it lets each profile take as much transparency as it can use. The interface shows a baseline explanation by default and keeps deeper reasoning accessible on demand (Brennen, 2020; Doshi-Velez and Kim, 2017; Ehsan et al., 2024). Cai et al. (2019) found that clinicians worked more effectively with layered explanations than with either full technical detail or no explanation, and that on-demand depth supported more accurate trust calibration. Not every user wants a full reasoning trace. Progressive disclosure manages cognitive load while preserving interpretability for those who seek it (Doshi-Velez and Kim, 2017).

Taken together, the five strategies offer a toolkit for narrowing the gap between reliability and interpretability, but they share a limitation that the typology makes visible. Every strategy described above waits for the user to act. A citation must be clicked, a rationale must be read, an uncertainty cue must be noticed. These affordances therefore serve the motivated profiles well and barely touch the unmotivated ones, who accept fluent outputs quickly and move on (Stanovich and West, 2000). Research on misinformation interventions points to the same conclusion, since participatory engagement changes thinking more durably than passive warnings (Kozyreva et al., 2024; Lim and Perrault, 2023). What remains is the harder question of how to give unmotivated users a reason to engage at all. The next section turns to that question.

## 6 Engagement-based incentive mechanisms: beyond passive transparency

The strategies in the previous section share a limitation: they make evaluation possible, but they still wait for the user to initiate the checks. Engagement-based incentive mechanisms remove this dependence by building evaluative moments into the flow of interaction itself. This is a shift from transparency as disclosure to transparency as practice. The question is now how the interaction should apply the user’s time and effort. Two lines of evidence suggest that engagement drives durable change, and that information alone does not. First, research on misinformation finds that active practices such as lateral reading, accuracy reflection, and inoculation games outperform passive warnings in lasting effect (Badrinathan, 2021; Roozenbeek and van der Linden, 2019; Traberg et al., 2022; Wineburg and McGrew, 2019). Second, research on human–LLM dialogue points the same way. Analyzing 397 human–LLM conversations about political issues, Furniturewala et al. (2026) found that the effect of the model’s explanatory richness on knowledge gain operated entirely through users’ cognitive engagement during the dialogue, and that longer conversations produced knowledge gains only for users with greater reflective and motivational resources. The design implication is that mechanisms must work with the appeal of fluent interaction rather than against it, or users will simply route around them.

Accuracy nudges (Pennycook et al., 2020), friction-based interventions (Kozyreva et al., 2024), commitment devices (Cialdini, 2009), and social proof cues (Celadin et al., 2024; Prike et al., 2024) all come from research on misinformation and behavior change. Our contribution is to transfer this logic to conversational AI and to show that the transfer changes what the interventions can do. Three features of the chatbot setting matter: first, that the interaction is sustained, so the system can observe behavior within a session, such as how quickly a user accepts answers. Second, that it is personalized, so the dose of an intervention can be adjusted to the individual. Third, that it is bidirectional, so a nudge can take the form of a question instead of a label. Static platforms offer none of these options. Mechanisms such as adaptive friction and engagement gates therefore have no equivalent in a social media feed, and studying them can tell us something the nudging literature so far could not.

Human–computer interaction research on AI-assisted decision making offers a nearer precedent, and the mechanisms below build on it. Buçinca et al. (2021) showed that cognitive forcing functions, such as delaying the AI’s recommendation or asking the user to answer before seeing it, reduce overreliance more effectively than explanations do, though at a measurable cost to satisfaction. Related work explains why forcing is needed at all. Explanations can increase acceptance of AI advice instead of scrutiny of it (Bansal et al., 2021), and they reduce overreliance only when engaging with them costs less than the benefit the user expects from checking (Vasconcelos et al., 2023; see Passi and Vorvoreanu, 2022, for a review). These studies establish that friction can work and that it is unevenly welcome.

In the following paragraphs, we offer ideas on interventions that are sustained, personalized, and bidirectional can calibrate trust, the underlying rationale, predictors, likely outcomes, and candidate target user profiles. Table 1 summarizes the framework.

**Table 1:** Six engagement mechanisms.

Mechanism	Trigger	Example implementation	Primary outcome measure	Primary profile served
Cooling-off period	High-stakes query, low model confidence, unverified acceptance streak	Brief delay with source preview before final response	Source click-through rate	High ability, low motivation
Engagement gate	High-stakes response before finalizing or export	Confidence rating or verification choice before copying	Completion vs. bypass rate	Both low-motivation profiles
Dialogue scaffold	Contested or multi-source topic	Alternative perspective offered before summary	Knowledge gain, counterargument quality	Low ability, high motivation
Adaptive friction	Behavioral pattern of uncritical acceptance	Graduated escalation from cue to prompt to gate	Verification change after escalation	Inferred from behavior
Social verification signal	Topic with high past verification or dispute	“Most users who asked this checked the sources”	Source click rate with vs. without signal	Low ability
Commitment prompt	Session end or answer export	“What would you verify before using this?”	Stated confidence vs. actual verification gap	Low motivation

### 6.1 Cooling-off periods

A cooling-off period inserts a brief delay before the chatbot delivers its final response or before the user can act on it, for example by copying or exporting. This mechanism builds on evidence that a short pause before judgment reduces the sharing of false content (Fazio, 2020). The trigger should be selective rather than universal. Candidate conditions include queries classified as high stakes, responses with low internal confidence, and a streak of accepted answers without any verification behavior. During the delay, the interface can show a source preview, which turns waiting time into checking time. Success is measurable as the source click-through rate during and after the delay. The mechanism is aimed at users with high ability but low motivation, because it interrupts the autopilot that their expertise otherwise excuses.

### 6.2 Engagement gates

An engagement gate asks the user to perform one evaluative action before a response is finalized or exported. The chatbot may deliver a preliminary answer and ask which aspect it should verify before finalizing, or it may ask users to rate their confidence in an answer before copying it. Gates operationalize the finding that dialogic engagement, and not information delivery, drives cognitive change (Furniturewala et al., 2026). Gates should activate under the same high-stakes conditions as cooling-off periods, and they must remain skippable to protect autonomy. Success is measurable by comparing completion rates with bypass rates, and by testing whether gated answers are verified more often. Gates serve both low-motivation profiles, because they supply the evaluative moment those users would not create themselves.

### 6.3 Structured dialogue scaffolds

A dialogue scaffold structures the conversation so that evaluation happens inside it. The chatbot may offer an alternative perspective before summarizing, show the strongest and the weakest of its sources and ask which the user finds more convincing, or ask what the user already believes before answering. Inoculation research supports this design, since generating counterarguments protects belief accuracy better than receiving conclusions (McGuire, 1964; Roozenbeek and van der Linden, 2019). Scaffolds fit topics that are contested or rest on multiple sources, and they are the clearest use of the bidirectional advantage described above. Success is measurable through knowledge gain and through linguistic markers of reflective engagement during the dialogue (Furniturewala et al., 2026). Scaffolds work best for users with high motivation but low ability, because they convert the willingness to check into an effective procedure for checking.

### 6.4 Adaptive friction based on interaction patterns

Adaptive friction monitors interaction patterns and adds resistance only when the pattern suggests uncritical acceptance. Signals include rapid acceptance of consecutive answers, absence of follow-up queries, and copy-paste behavior without any evaluative action. The response escalates gradually, from highlighting uncertainty in the next answer, to an explicit prompt that names the pattern, to a short gate. This mechanism extends adaptive nudging (Kozyreva et al., 2024) and the idea of desirable difficulties in learning (Bjork and Bjork, 2011). This mechanism is the purest product of the conversational setting, because it requires the within-session behavioral data that only sustained interaction provides. Success is measurable as the change in verification behavior after each escalation step. Adaptive friction is not tied to one profile. It infers the profile from behavior and doses itself accordingly.

### 6.5 Social verification signals

A social verification signal shows the user how others handled the same content. Examples include a note that most users who asked this question also checked the sources, or a flag that expert users frequently mark this topic for verification. These signals draw on social proof and norm-based interventions, which have improved truth discernment and reduced engagement with misinformation in several studies (Koch et al., 2023; Prike et al., 2024), although a direct tournament of message-based norm nudges found no significant effects (Celadin et al., 2024), so this mechanism in particular must earn its keep empirically. The trigger is content-based, not behavior-based, activating on topics with high rates of past verification or dispute. Success is measurable as the source click rate with and without the signal. These signals help low-ability users most, because they replace a check the user cannot perform with a cue the user can read.

### 6.6 Commitment and reflection prompts

A commitment prompt asks the user to state a position before leaving. The chatbot may ask how confident the user is in the answer, or what one thing they would verify before using it. Such prompts combine commitment and consistency effects from persuasion research (Cialdini, 2009) with the accuracy-nudge paradigm (Pennycook et al., 2020). The trigger is the end of a session or the export of an answer, which makes the prompt cheap to deliver and hard to miss. Success is measurable as the gap between stated confidence and later verification, and as reduced unverified reuse of chatbot content. These prompts serve low-motivation users, because a stated commitment creates a reason to check that fluency alone never supplies.

### 6.7 Design principles for engagement mechanisms

Five principles govern how these mechanisms should behave in use. Friction must be proportional, so that low-stakes queries stay frictionless while high-stakes queries carry more. Mechanisms must be adaptive, because the typology predicts that a dose useful for one profile is an annoyance for another, and users who already verify should meet less friction rather than more. Friction must be transparent, since a system that explains why it slows down builds trust, while unexplained friction reads as malfunction. Mechanisms must remain non-paternalistic, so that users can always override them; the goal is to support judgment, not to ration information. Finally, every mechanism must be treated as an empirical hypothesis. Its effects on calibration, accuracy, and satisfaction are testable, and we state formal predictions to that end below.

These principles can be stated as one operating rule, which also answers how a system should behave when it cannot know the user in advance. Interpretability affordances stay on for everyone, because they cost the user nothing until the user chooses to act on them. Friction is off by default. It is triggered only when two conditions hold at once, namely that the query is high stakes and that recent behavior suggests uncritical acceptance. The system then escalates in small steps and stops at the first step that produces verification behavior. When a user verifies without being prompted, the friction is withdrawn again. Every step remains skippable, and a rising skip rate is the signal that the dose has gone too far. A system that follows this rule adapts to the profile without having to measure it.

**Figure 2:**
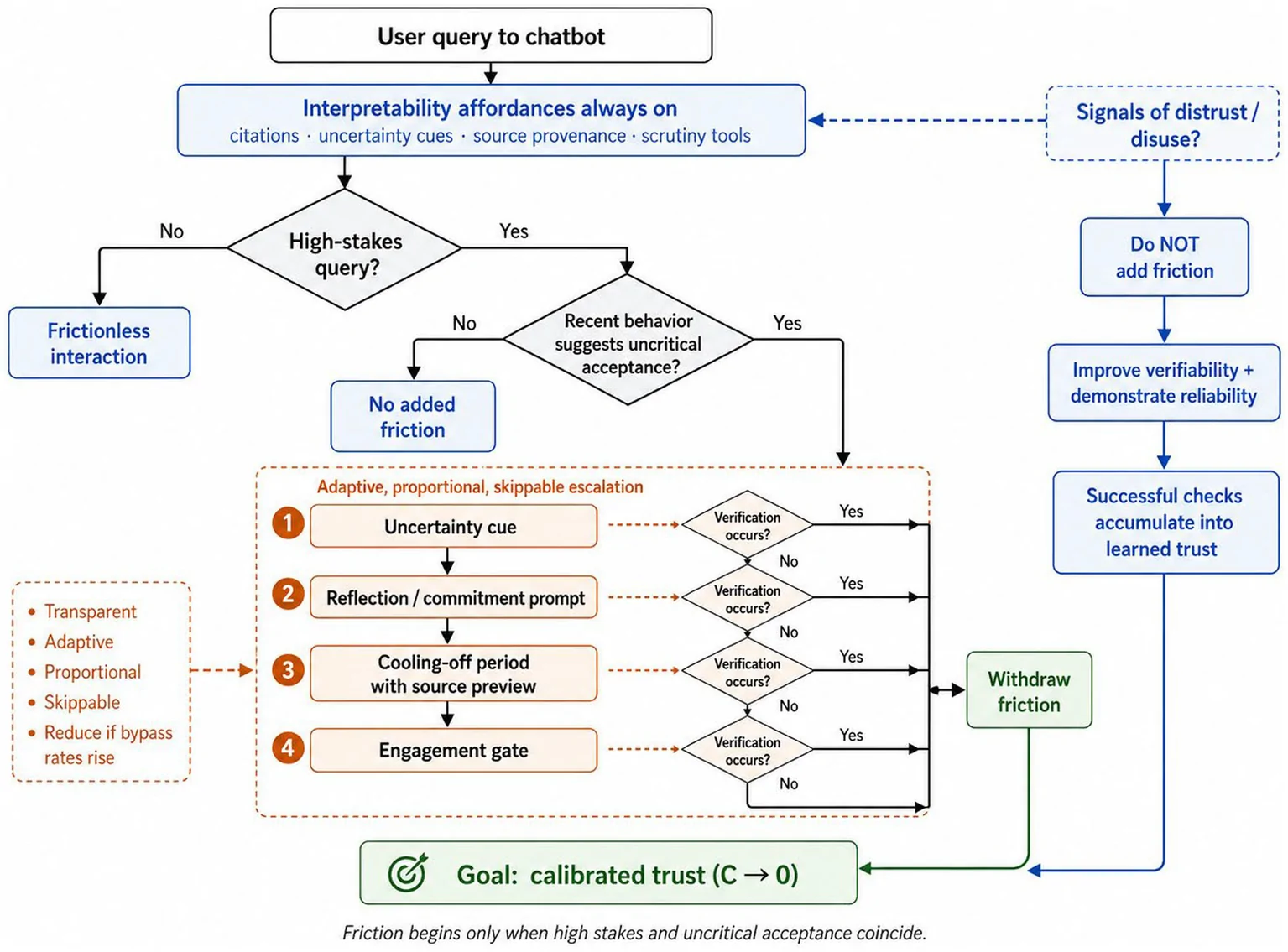
Adaptive design logic for trust calibration. Interpretability affordances remain available throughout the interaction, whereas friction is introduced selectively when a high-stakes query coincides with behavioral signs of uncritical acceptance. Friction escalates in small, skippable steps and is withdrawn when verification occurs. For users showing distrust or disuse, the framework predicts the opposite response: reducing friction and improving verifiability so that successful checks can accumulate into learned trust.

Figure 2 translates this operating rule into a candidate adaptive design sequence. Rather than assigning users permanently to a profile, the system responds to the stakes of the query and to observable interaction behavior, adding friction only when both indicate a risk of uncritical acceptance.

## 7 A Swiss cheese model of trust calibration

Taken together, these mechanisms and the affordances of the previous section form a layered defense, which Reason’s (1990) Swiss cheese model describes well. In the original model, every safety barrier has holes, yet stacked barriers rarely let a hazard through all of them at once. The model reached a wide public during the COVID-19 pandemic, when masks, ventilation, and vaccines were each imperfect and jointly effective. Chatbot design can borrow the same logic, and the typology tells us where each layer’s holes are. Interpretability affordances miss unmotivated users. Engagement mechanisms catch them, but may miss users whose checks are ineffective. Literacy building, which the next section discusses, closes gaps slowly but durably. A user who cannot evaluate citations slips through source transparency, yet a dialogue scaffold can still engage that user, and a social signal can still redirect them. No single intervention can fully recalibrate user trust. Layered combinations can make uncritical acceptance rare.

### 7.1 Testable propositions

The framework developed above yields propositions that combine the two dimensions of the user typology with the two families of design intervention. The predictions are quantitative. At the outset, we define the calibration gap C as the mean confidence a user reports minus the proportion of answers that are correct. A C of zero means calibrated trust, a positive C means overtrust, and a negative C means distrust. In the propositions below, an improvement means a move toward zero, and a gain is the size of that move relative to a control condition. Comparisons across profiles are therefore claims about interactions, not about main effects. Behavioral measures, such as how often users check a source or whether they accept correct answers and reject incorrect ones, can serve the same purpose.

The first two propositions concern the division of labor between the two families of intervention.

*Proposition 1*. *Interpretability affordances improve trust calibration among users with high motivation to verify, and have little effect among users with low motivation*. Every affordance waits for the user to act, so its benefit is conditional on the will to act.

*Proposition 2*. *For low-motivation users, engagement mechanisms improve calibration more than interpretability affordances do*. The mechanisms create the evaluative moment that these users do not create for themselves.

The next three propositions locate specific mechanisms within the typology.

*Proposition 3. Cooling-off periods and adaptive friction produce their largest gains among users with high ability and low motivation, and time pressure amplifies the effect*. These users can evaluate an answer once something interrupts their autopilot, so a pause converts existing ability into actual checking. The proposition also settles a practical question. A cooling-off period is not for the novice who lacks knowledge but for the expert who lacks the moment.

*Proposition 4. Structured dialogue scaffolds produce their largest gains among users with high motivation and low ability*. Scaffolds supply the procedure that motivated but inexpert checking lacks. Their benefit should therefore shrink as expertise grows.

*Proposition 5*. Social verification signals produce their largest gains among low-ability users, regardless of motivation. The signal substitutes a readable cue for a check the user cannot perform. Its value falls as the user’s own verification becomes reliable.

The next two propositions concern dosage and layering.

*Proposition 6*. Friction *beyond the calibration point backfires.* Among users whose trust is already well calibrated, added friction reduces satisfaction first and use second, and pushes the best calibrated users toward disuse. A framework that predicts only successes explains too little. This proposition marks where the interventions should stop. On this scale, it predicts a move past zero rather than a smaller gain.

*Proposition 7*. *Combinations of interventions from different layers outperform combinations of equal size from the same layer.* Two affordances share the same hole, since both miss unmotivated users. An affordance paired with an engagement mechanism covers each other’s misses. This is the Swiss cheese model stated as a prediction.

A final proposition addresses the direction of miscalibration that the engagement mechanisms leave untreated.

*Proposition 8. Among distrusting users, interpretability affordances paired with demonstrated reliability move C toward zero, while added friction moves it further away.* Disuse is a rational response when verification is impossible, so the remedy is to make verification possible and let successful checks accumulate into learned trust (Hoff and Bashir, 2015). Friction, by contrast, raises the cost of an interaction these users already undervalue. The proposition marks the second boundary of the engagement family. Its mechanisms treat overtrust, and they have no role in treating distrust.

While these propositions are not exhaustive, they offer ways to validate it. While they should hold on both confidence and behavior, a result that appears on one but not the other is itself informative. The tests will require a measurement of ability and motivation alongside behavior, for example through domain knowledge tests, need for cognition scales (Cacioppo and Petty, 1982), and logged verification actions. Two limits of the stated operationalizations deserve notice, in the spirit of the warning from Vereschak et al. (2021) about mismatches between definitions and measures. First, C records confidence, whereas trust as defined in this article is a willingness that shows itself in reliance. Studies should therefore log verification behavior alongside C and treat the two as the attitudinal and behavioral readings of the same construct. Second, C is a mean-level index. A user can score zero while being confident in wrong answers and doubtful of correct ones. Discrimination measures complement it, such as the rate of appropriate reliance, defined as accepting correct answers and rejecting incorrect ones, and calibration curves across confidence levels.

## 8 Outlook: building literacy as the durable layer of defense

User literacy is the third layer of the defense described in this article, and it works differently from the other two. While affordances and engagement mechanisms take users as they are, routing around low motivation or low ability, literacy aims to change the user. It raises the ability to verify, and often the motivation with it, so it moves people across the profiles of our typology instead of working within them. This is why literacy is the slowest layer to build and the most durable once built.

The literacy that matters here is specific to AI. Conventional digital literacy teaches users to evaluate sources and detect bias in human-authored content. AI literacy adds an understanding of how generative systems work, including their probabilistic nature, their tendency to fabricate, and the meaning of their transparency cues (Long and Magerko, 2020; Ng et al., 2021). Trust in AI is not a fixed attitude but something shaped through use (Jacovi et al., 2021). A user who knows why a model hallucinates reads an inline citation differently from a user who does not.

Existing frameworks and programs give reason for measured optimism. The AILit framework developed by the European Commission and the OECD spans engaging with, creating with, managing, and designing AI (World Economic Forum, 2025). Long and Magerko (2020) specify 17 competencies, and Ng et al. (2021) place critical evaluation and ethics alongside functional skill. Training built on such models improves discernment and reduces overreliance (Digital Education Council, 2024). Teaching lateral reading improves source evaluation (Wineburg and McGrew, 2019), and a brief tips-based digital literacy intervention improved discernment between mainstream and false news in the United States and India (Guess et al., 2020). The caution concerns transfer. Breakstone et al. (2021) found that students who had received standard digital literacy instruction still failed to distinguish reliable from unreliable sources in practice. Generic media literacy does not automatically transfer to AI-generated content, which is more fluent and carries fewer warning marks than the content those curricula were built for.

When literacy interventions do work, they last. Media literacy and inoculation approaches consistently outperform one-off corrections in producing durable resistance to misleading content (Badrinathan, 2021; Lu et al., 2024; McGuire, 1964; Traberg et al., 2022). Durability is exactly what the other two layers lack, as an affordance stops working the moment the user switches platforms. On the contrary, a verification habit travels with the user.

The engagement mechanisms proposed in this article can serve as literacy interventions in miniature. A dialogue scaffold that asks the user to weigh two sources teaches source comparison through practice. A commitment prompt that asks what the user would verify rehearses a habit. Embedded in daily use, these mechanisms train critical engagement in places that instruction alone does not reach (Agley et al., 2021; Hofer and Pintrich, 1997). The moments must be designed deliberately, however, because left alone, efficiency wins. Gow and Illingworth (2026) found that students framed AI overwhelmingly in terms of productivity, not learning.

Deliberate design, in turn, presupposes a deployer, and a framework of this kind must say who that would be. Friction runs against the engagement metrics that govern consumer platforms, and adaptive friction requires behavioral monitoring that creates privacy obligations of its own, since the signals that dose an intervention could also profile a user. Behavioral data used for dosing should therefore remain within the session where possible and serve no secondary purpose. Deployment need not depend on platform goodwill, however. Regulation can mandate transparency and risk-proportionate design, as the European Union’s AI Act begins to do for systems it classes as high risk. Institutional procurement can demand calibration-supporting features in education, health, and government settings, where the buyer is not the platform and the cost of miscalibration falls on the institution. The mechanisms themselves supply the evidence that both routes require, because each one carries a measurable outcome.

Calibrated trust in chatbots is therefore a shared product. Designers must build systems that afford interrogation and invite engagement. Educators must prepare users who can read what those systems show. Regulators and institutional buyers must create the conditions under which such systems get built at all. No party can fully compensate for the others, which is the Swiss cheese model’s final lesson. As LLM-based systems settle into the infrastructure of public knowledge, the goal is not more trust, and not less. The goal is trust that matches what these systems actually deserve, user by user and answer by answer.

## Data Availability

The original contributions presented in the study are included in the article/supplementary material, further inquiries can be directed to the corresponding author.
